# Identification of Novel Amelogenin-Binding Proteins by Proteomics Analysis

**DOI:** 10.1371/journal.pone.0078129

**Published:** 2013-10-22

**Authors:** Takao Fukuda, Terukazu Sanui, Kyosuke Toyoda, Urara Tanaka, Takaharu Taketomi, Takeshi Uchiumi, Fusanori Nishimura

**Affiliations:** 1 Department of Periodontology, Division of Oral Rehabilitation, Faculty of Dental Science, Kyushu University, Fukuoka, Japan; 2 Dental and Oral Medical Center, Kurume University School of Medicine, Kurume, Fukuoka, Japan; 3 Department of Clinical Chemistry and Laboratory Medicine, Graduate School of Medical Sciences, Kyushu University, Fukuoka, Japan; New York University, United States of America

## Abstract

Emdogain (enamel matrix derivative, EMD) is well recognized in periodontology. It is used in periodontal surgery to regenerate cementum, periodontal ligament, and alveolar bone. However, the precise molecular mechanisms underlying periodontal regeneration are still unclear. In this study, we investigated the proteins bound to amelogenin, which are suggested to play a pivotal role in promoting periodontal tissue regeneration. To identify new molecules that interact with amelogenin and are involved in osteoblast activation, we employed coupling affinity chromatography with proteomic analysis in fractionated SaOS-2 osteoblastic cell lysate. In SaOS-2 cells, many of the amelogenin-interacting proteins in the cytoplasm were mainly cytoskeletal proteins and several chaperone molecules of heat shock protein 70 (HSP70) family. On the other hand, the proteomic profiles of amelogenin-interacting proteins in the membrane fraction of the cell extracts were quite different from those of the cytosolic-fraction. They were mainly endoplasmic reticulum (ER)-associated proteins, with lesser quantities of mitochondrial proteins and nucleoprotein. Among the identified amelogenin-interacting proteins, we validated the biological interaction of amelogenin with glucose-regulated protein 78 (Grp78/Bip), which was identified in both cytosolic and membrane-enriched fractions. Confocal co-localization experiment strongly suggested that Grp78/Bip could be an amelogenin receptor candidate. Further biological evaluations were examined by Grp78/Bip knockdown analysis with and without amelogenin. Within the limits of the present study, the interaction of amelogenin with Grp78/Bip contributed to cell proliferation, rather than correlate with the osteogenic differentiation in SaOS-2 cells. Although the biological significance of other interactions are not yet explored, these findings suggest that the differential effects of amelogenin-derived osteoblast activation could be of potential clinical significance for understanding the cellular and molecular bases of amelogenin-induced periodontal tissue regeneration.

## Introduction

Amelogenins (enamel matrix proteins) are a group of low-molecular-weight proteins found in developing tooth enamel [1]; it belongs to a family of extracellular matrix (ECM) proteins. Amelogenin genes are highly conserved in vertebrates [2], and this stability indicates the essential role in enamel formation. Amelogenin-encoding *Amelx* gene is located within the first intron of *Arhgap6* gene on the X-chromosome [3]. In humans, mutations of *AMELX* gene lead to X-linked amelogenesis imperfecta (AI) [4]. 

The process of cementum deposition is a prerequisite for the formation of periodontal ligament and alveolar bone. The role of amelogenins in periodontal ligament formation is supported by their presence during the development of cementum by directing the cells that form cementum to the root surface of teeth [5]. During tooth development, amelogenins are secreted mainly by ameloblasts and partly by Hertwig's epithelial root sheet (HERS) cells [6]. Amelogenin is temporarily deposited onto the dentin root surface, and form an initial and essential step in cementogenesis [7]. Previous *in vivo* studies have revealed that amelogenin null mice show abnormal resorption of cementum [8]. Thus, amelogenin not only plays an important role in enamel formation, but also as a mediator of cementogenesis and in the attachment of periodontal ligament.

To date, a variety of periodontal regenerative therapies has been developed [9], and the administration of ECM is one of the ideal therapeutic strategy [10]. Based on this biomimetic strategy that tries to mimic the events during the teeth development process [11], enamel matrix derivative (EMD) (Straumann® Emdogain) is widely used for periodontal tissue regeneration and the long-term clinical results appear to be stable [12]. A number of studies have demonstrated the osteoconductive activity of EMD, in particular, for human periodontal ligament cells and osteoblastic cell types [13]. EMD enhances osteoblast differentiation and mineralization [14], as well as contributes to multi-lineage differentiation of human periodontal ligament cells [15]. Furthermore, transplantation of induced pluripotent stem cells combined with EMD greatly enhanced periodontal tissue regeneration [16].

Amelogenin, the major component of enamel matrix proteins, is suggested to be a bioactive candidate for periodontal regeneration [11,17]; however, that does not exclude the possibility that other components of the enamel matrix proteins also contribute to the regeneration process [18]. Nevertheless, recent studies have shown that recombinant amelogenin alone stimulates osteogenic differentiation of mesenchymal stem cells [19], as well as promotes regeneration of bone and periodontal tissues [20]. Despite the significant role of amelogenin in the EMD-induced regeneration process of the periodontium, the precise downstream targets and potential modulators of this signaling adaptor have not been well defined as yet. This may be mainly because of the difficulties in the isolation of amelogenin-targeting proteins. In addition, no studies have reported the protein interaction of amelogenin in cells that participate in periodontal regeneration. Based on the fact that amelogenins are secreted mainly by ameloblasts during teeth development, only ameloblast cells were examined for the identification of amelogenin-binding proteins by yeast two-hybrid assay [21].

In the present study, therefore, we combined affinity chromatography and proteomic analysis to identify amelogenin binding proteins in osteoblastic cells. Herein, we report the characterization of newly identified amelogenin-binding molecules that could be candidates for amelogenin induced periodontal tissue regeneration.

## Materials & Methods

### Ethics Statement

All procedures using mice were performed in strict accordance with the guidelines for Proper Conduct of Animal Experiments (Science Council of Japan). They were housed in temperature- and light-controlled environmental conditions with a 12-hour light and dark cycle, and permitted ad libitum consumption of water and standard pellet chow. The experimental protocol was approved by the Animal Care and Use Committee of Kyushu University (Permit Number: A22-130-2). All efforts were made to minimize the number of animals used and their suffering.

### Cell Culture and Subcellular Protein Extraction

The human osteoblastic cell line SaOS-2 was obtained from the Riken Cell Bank (Tsukuba, Japan). SaOS-2 cells were cultured in growth medium (GM) consisting of alpha-minimum essential medium (Gibco-BRL, Grand Island, NY), supplemented with 50 μg/mL streptomycin, 50 U/mL penicillin (Gibco-BRL), and 10% fetal bovine serum (Gibco-BRL), at 37°C in a 5% CO2 incubator.

For osteogenic differentiation, SaOS-2 cells at confluence were cultured in osteogenic medium (OM) consisting of GM supplemented with 2 mM β-glycerophosphate (Wako, Japan) and 50 μg/mL ascorbic acid (Wako).

Total soluble protein was extracted using the Cytobuster Protein Extraction Reagent (Novagen, Madison, WI) supplemented with 10 µL/mL of Protease Inhibitor Cocktail for Use with Mammalian Cell and Tissue Extracts that consisted of (4-(2-aminoethyl) benzenesulfonyl fluoride (AEBSF), pepstatinA, E-64, bestatin, leupeptin hemisulfate monohydrate and aprotinin (Nakarai Tesque, Japan). Membrane proteins were isolated using the ProteoExtract™ Native Membrane Protein Extraction kit (Calbiochem, Temecula, CA) according to the manufacturer’s protocol.

### Cloning, Expression, and Purification of Recombinant Amelogenin

Mice were mated overnight, and females were examined for a vaginal plug the following morning. At noon of that day, vaginal plug detection was recorded as embryonic day (E) 0.5. Total RNA was prepared from the entire head of fetal C57BL/6 mouse at E18.5; this developmental stage offers maximum expression of amelogenin cDNA isoform M180 [22]. Full-length cDNA for mouse amelogenin (M180) was amplified by reverse transcription-polymerase chain reaction using the following primer pair (initiation codon is underlined): M180F, 5′-AAAGGATCCATGCCCCTACCACCTCATCCT-3′ and M180R 5′-TTTCTCGAGTTAATCCACTTCTTCCCGCTT-3′. M180 was cloned into the *Bam*HI/*Xho* I sites of the pGEX-6P vector (Amersham Pharmacia Biotech, Piscataway, NJ) for purification of a glutathione S-transferase (GST) fusion protein as reported previously[23]. Plasmids coding for the protein of interest were transformed into competent *Escherichia coli* (BL21 DE3-RIl) (Stratagene, La Jolla, CA), according to the manufacturer’s protocol, and grown to an optical density of 0.6–0.8 (*A*_600_) at 30°C. Expression was induced with 0.1 mm isopropyl β-d-thiogalactoside (Fisher Scientific, Pittsburgh, PA) and cells were grown for 5 h at 25°C. Post-expression cultures were centrifuged at 8,000 × *g* for 15 min at 4°C; bacterial pellets were stored at −80°C prior to protein purification.

The bacterial pellets containing recombinant GST-rM180 were resuspended in 1 mL of B-PER^®^ Bacterial Protein Extraction Reagent (Pierce), 1 mM DTT, and Protease Inhibitor Cocktail (Nakarai Tesque, Japan). The resuspended pellets were incubated on ice for 30 min. The pellets were subsequently sonicated (Astrason Ultrasonic Processor) on ice to obtain complete lysis of the bacteria. Cell extracts were purified by centrifugation for 15 min at 20,000 × *g* at 4°C, and approximately 100 µg of GST-rM180 was bound to 100 µL of glutathione-Sepharose 4B beads (GE Healthcare Life Sciences) for 1 h at 4°C. The beads were transferred to Micro Bio-Spin chromatography columns (Bio-Rad), washed with a total of 10 mL of washing buffer (50 mm Tris-HCl, pH 7.5, 150 mm NaCl, 10% glycerol, and 0.2% Triton X-100), and then with 1 mL of PreScission buffer (50 mm Tris-HCl, pH 8.0, 150 mm NaCl, 1 mM EDTA, 1mM DTT) for purification. On-column cleavage of rM180 from the GST portion of the fusion protein was carried out with the PreScission protease (GE Healthcare) according to the manufacturer’sprotocol.

Protein samples were loaded on a 10% sodium dodecyl sulfate (SDS)-polyacrylamide gel. The gels were stained with Coomassie brilliant blue (CBB Stain One; Nakarai, Osaka, Japan) and inspected visually for protein expression. After separation via SDS- polyacrylamide gel electrophoresis (SDS-PAGE), western blot analysis was performed with anti-amelogenin antibody (F-11) (1:1000; Santa Cruz Biotechnology, Inc., Santa Cruz, CA).

### Monitoring Endocytosis of Amelogenin and Co-localization with Grp78/Bip

SaOS-2 cells (6 × 10^4^ cells per well) were grown on glass coverslips placed in 12-well tissue culture dishes in α-MEM containing 10% fetal bovine serum for at least 24 h. For the time course experiments, SaOS-2 cells were incubated with 30 µg/ml rM180 for 60 min at 4°C before incubation for the indicated periods of time at 37°C. The cells were then extensively washed with phosphate-buffered saline, fixed with 4% paraformaldehyde, permeabilized with 50 μg/mL digitonin, and blocked with 3% bovine serum albumin in phosphate-buffered saline. Fluorescence detection of amelogenin was performed with a mouse monoclonal anti-amelogenin primary antibody (F-11) (1:250; Santa Cruz Biotechnology, Inc., Santa Cruz, CA) and a goat anti-mouse Alexa Fluor 488 secondary antibody (1:1000; Molecular Probes, Invitrogen, Carlsbad, CA). To examine co-localizaion of rM180 with Grp78/Bip, goat polyclonal anti-Grp78/Bip primary antibody (C-20) (1:250; Santa Cruz Biotechnology, Inc., Santa Cruz, CA) and a donkey anti-goat Alexa Fluor 594 secondary antibody (1:1000; Molecular Probes, Invitrogen, Carlsbad, CA) were also used. Nuclei were stained with Hoechst 33258 (10 µg/mL; Molecular Probes, Invitrogen, Carlsbad, CA). The coverslips were mounted using PermaFluor Mounting medium (Thermo Fisher Scientific, Waltham, MA). Cells were visualized under a Nikon A1 fluorescence microscope.

### Combination of Affinity Chromatography and Proteomic Analysis

The GST pull-down assay was performed by following our standard protocol [24]. The bacterial lysates containing recombinant GST and GST-rM180 were immobilized (50 µg each) on glutathione-Sepharose 4B beads (GE Healthcare) for 1 h at 4°C, and centrifuged for 5 min at 500 × *g* at 4°C to pellet the beads. Meanwhile, the fractionated SaOS-2 cell lysates were pre-incubated with glutathione-resin without any GST fusion proteins for 1 h at 4°C. The beads were washed with a total of 5 mL of washing buffer. To pull down rM180-binding proteins, purified GST-rM180 immobilized on glutathione-resin was incubated with 10 mg of the fractionated protein extracts from SaOS-2 cells for 1-hour at 4°C with gentle rotation. The beads were transferred to spin columns and washed with a total of 5 mL of washing buffer, followed by1 mL of phosphate-buffered saline. Total bound proteins were eluted with 150 µL of rehydration buffer (8 M urea, 2% 3-((3-cholamidopropyl)dimethylammonio)-1-propanesulfonic acid, 50 mM DTT, 0.2% Bio lyte 3–10 ampholytes, and a trace of bromophenol blue dye) (Bio-Rad, Hercules, CA, USA) and subjected to two-dimensional PAGE (2D-PAGE). 

2D-PAGE was used to separate the cellular proteins as previously described [25]. Prior to 2D-PAGE, contaminants including lipids, salts, and detergents were removed from the affinity-purified GST protein samples using ReadyPrep™ 2D Clean-Up Kit (Bio-Rad, Hercules, CA, USA). This kit is based on TCA/acetone precipitation of proteins. After precipitation, the purified protein samples were washed and then resuspended in the rehydration buffer (Bio-Rad). The purified protein samples, diluted with 125 µL of rehydration buffer (Bio-Rad), were loaded onto an isoelectric focusing (IEF) strip (7 cm, pH 3–10; BioRad) in a PROTEAN IEF Cell (Bio-Rad). The strip was covered with mineral oil and rehydrated for 18 h at 50 V at 20°C. IEF was then carried out at 250 V for 20 min, 4,000 V for 2.5 h, and then 4,000 V for 10 KV/h. After focusing, the gel strip was incubated in fresh equilibration buffer 1 [6 M urea, 2% SDS, 0.375 M Tris–HCl, 20% glycerol, and freshly made 2% DTT] and equilibration buffer 2 (6 m urea, 2% SDS, 0.375 m Tris–HCl, 20% glycerol, and freshly made 2.5% iodoaceamide) for 10 min each with shaking. The IEF strips were embedded on top of 20% polyacrylamide gradient gels (BioRad) using 0.5% (weight/volume) molten agarose. SDS-PAGE was performed at 120 V for 100 min at room temperature. The gels were stained with Coomassie brilliant blue (CBB Stain One; Nakarai, Osaka, Japan).

Matrix-assisted laser desorption/ionization time-of-flight (MALDI-TOF) mass spectrometry (MS) analysis of the amelogenin-binding proteins was performed using an Applied Biosystems 4700 proteomics analyzer at Genomine, Inc. (Pohang, Korea). Both MS and MS/MS data were acquired, and the MASCOT program (http://www.matrixscience.com) was used for sequence tag searches.

### Knockdown Analysis using Small Interfering RNA (siRNA)

Stealth^TM^ RNAi duplexes against human Grp78/Bip, which is a mixture of three different siRNAs (HSS105076, HSS105077, and HSS179390; GC content are 52%, 52%, and 48%, respectively) were obtained from Invitrogen Corporation (Invitrogen Life Technologies, Carlsbad, CA). All the Stealth™ RNAi sequences were blasted against the human genome database to eliminate cross-silencing phenomena with non-target genes, and to ensure the specificity of Grp78/Bip gene as the only target. As control, we utilized a Stealth^TM^ RNAi negative control duplex (Medium GC Duplex, Invitrogen Life Technologies) with a GC content of 48%, suitable for use as a control with Stealth^TM^ RNAi duplexes of 45–55% GC content. siRNA transfections were performed according to the manufacturer's reverse transfection protocol (Invitrogen Life Technologies). Briefly, 3 μL of Lipofectamine™ 2000 (Invitrogen Life Technologies) was diluted in 50 μL of Opti-MEM I medium (Invitrogen Life Technologies) and incubated for 5 min at room temperature (25°C). Next, 10 pmol of *Grp78/Bip* or control duplex Stealth RNAi in 50 μL of Opti-MEM I was added gently and incubated for 20 min at room temperature. Stealth^TM^ RNAi – Lipofectamine™ 2000 complexes and aliquots of 3 × 10^5^ SaOS-2 cells in 2 mL of culture medium were combined and incubated for 5 min at room temperature. The numbers of transfected cells were adjusted appropriately, and cell proliferation and osteoblast-specific gene expressions with and without the addition of rM180 were analyzed. 

### Cell proliferation assay

siRNA transfected SaOS-2 cells (5 × 10^3^ cells) in 100 µL of α-MEM containing 10% fetal bovine serum were seeded into each well of a 96-well culture plate in triplicate. The number of seeded cells was restrained to ensure that the cells could grow until the end of incubation period, without reaching cell confluence. The cells were incubated with 30 µg/mL rM180 after 24 h post transfection. Next, 10 μL of WST-8 solution (Cell Count Reagent SF™: Nakarai Tesuque, Kyoto, Japan) was added to each well, including the cell count control wells, at 24, 48, and 96 h. After additional 3 h of incubation at 37°C, optical absorption at 450 nm was measured with a reading at a wavelength of 650 nm as the reference.

### Total RNA Extraction for Reverse Transcription and Quantitative Real-time PCR (qRT-PCR)

Total RNA was extracted using the RNeasy^®^ Mini Kit and accompanying QIAshredder™ (Qiagen, Valencia, CA), according to the manufacturer's instructions. To avoid DNA contamination of the samples, column incubation with DNase I (Qiagen) was carried out for 15 min. Reverse transcription was performed using 500 ng of total RNA with a Superscript^®^ VILO^TM^ MasterMix (Invitrogen Life Technologies). The RT reaction mixtures were diluted at a ratio of 1:5 with water. qRT-PCR was performed with *Power* SYBR^®^ Green PCR Master Mix (Invitrogen Life Technologies) using an ABI Step 1 Plus Real-Time PCR System (Applied Biosystems, Life Technologies Corporation, Carlsbad, CA). Thermal cycling conditions consisted of an initial activation at 95°C for 10 min, followed by 40 cycles of two-step PCR; 95°C for 15 s, 60°C for 1 min, followed by a melt curve analysis. A correction was performed using a passive reference dye (Rox) present in the PCR master mix. The resulting data was recorded and analyzed using StepOne^TM^ software V2.2.2 (Applied Biosystems, Life Technologies Corporation) by selecting the auto calculated threshold cycle. To increase accuracy of the gene expression analysis, β-actin (ACTB) and glyceraldehyde-3-phosphate dehydrogenase (GAPDH) were used as multiple endogenous controls for the normalization of gene expression. When using multiple endogenous controls, the software treats all endogenous controls as a single population, and calculates the experiment-appropriate mean to establish a single value against which the target of interest is normalized. The cycle threshold (*C*_*T*_) values were determined, and mRNA expression levels were normalized to the multiple endogenous controls and expressed relative to the controls following the 2^−ΔΔCT^ method. The primer sequences used in this study are described in Table 1. 

**Table 1:** Primers used for qRT-PCR.

	**Forward primer**	**Reverse primer**
*human ALP*	5'-GACAAGAAGCCCTTCACTGC-3'	5'-AGACTGCGCCTGGTAGTTGT-3'
*human Osx*	5'-GGCACAAAGAAGCCGTACTC-3'	5'-GTAAAGGGGGCTGGATAAGC-3'
*human OCN*	5'-GGCGCTACCTGTATCAATGG-3'	5'-TCAGCCAACTCGTCACAGTC-3'
*human OPN*	5'-ACACATATGATGGCCGAGGTGA-3'	5'-TGTGAGGTGATGTGTCCTCGTCTGTAG-3'
*human Runx2*	5'-GCGTCAACACCATCATTCTG-3'	5'-CAGACCAGCAGCACTCCATC-3'
*human Col1*	5'-CCCGGGTTTCAGAGACAACTTC-3'	5'-TCCACATGCTTTATTCCAGCAATC-3'
*human ACTB*	5'-TGGCACCCAGCACAATGAA-3'	5'-CTAAGTCATAGTCCGCCTAGAAGCA-3'
*human GAPDH*	5'-ATCAAGAAGGTGGTGAAGCAGG-3'	5'-GTCATACCAGGAAATGAGC-3'

## Results

### Cellular uptake of amelogenin

**Figure 1:**
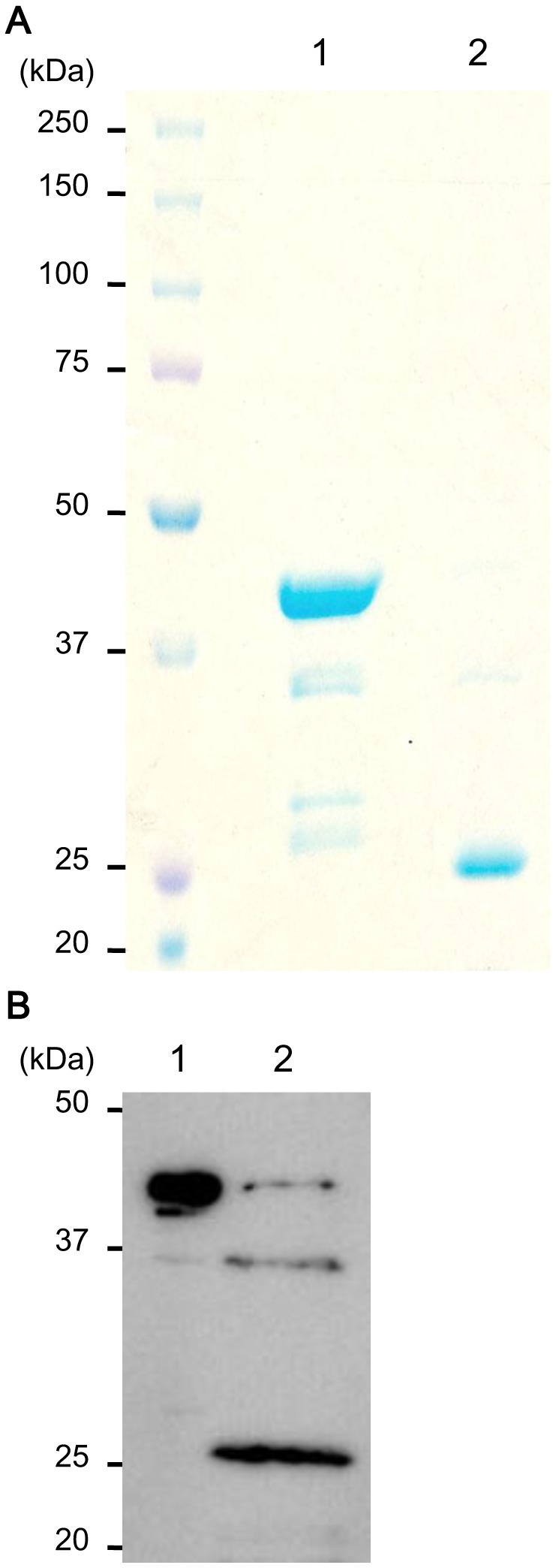
Generation of glutathione S-transferase (GST)-rM180 fusion proteins and purification of rM180 polypeptides. Sodium dodecyl sulfate-polyacrylamide gel electrophoresis (SDS-PAGE) visualization (**A**) and western blot detection (**B**) of GST-rM180 proteins (lane 1) and their cleaved products (lane 2), indicating purified rM180 polypeptides. The molecular weight markers are shown in kDa on the left of the gels. Proteins were visualized with Coomassie brilliant blue staining and confirmed with anti-amelogenin antibody.

As a precondition for establishing the physiological interaction between amelogenin and the cellular proteins, we firstly examined the pattern of cellular internalization of amelogenin. To verify endocytosis of amelogenin, rM180 was produced in *Escherichia coli* as GST fusion protein using the pGEX-6P expression system. The rM180 polypeptides were released from GST in a multiple step process, which involved binding of the fusion protein to glutathione-Sepharose, cleavage of rM180 fragment by prebound PreScission protease, and elution of the cleaved polypeptide. The purity of rM180 was tested by SDS-PAGE followed by CBB staining and was found to be >90% pure. Purified rM180 was detected as a single band of 25 kDa, as expected (Figure 1A). Identification of the recombinant protein was confirmed with western blot analysis using the anti-amelogenin antibody (Figure 1B).

**Figure 2:**
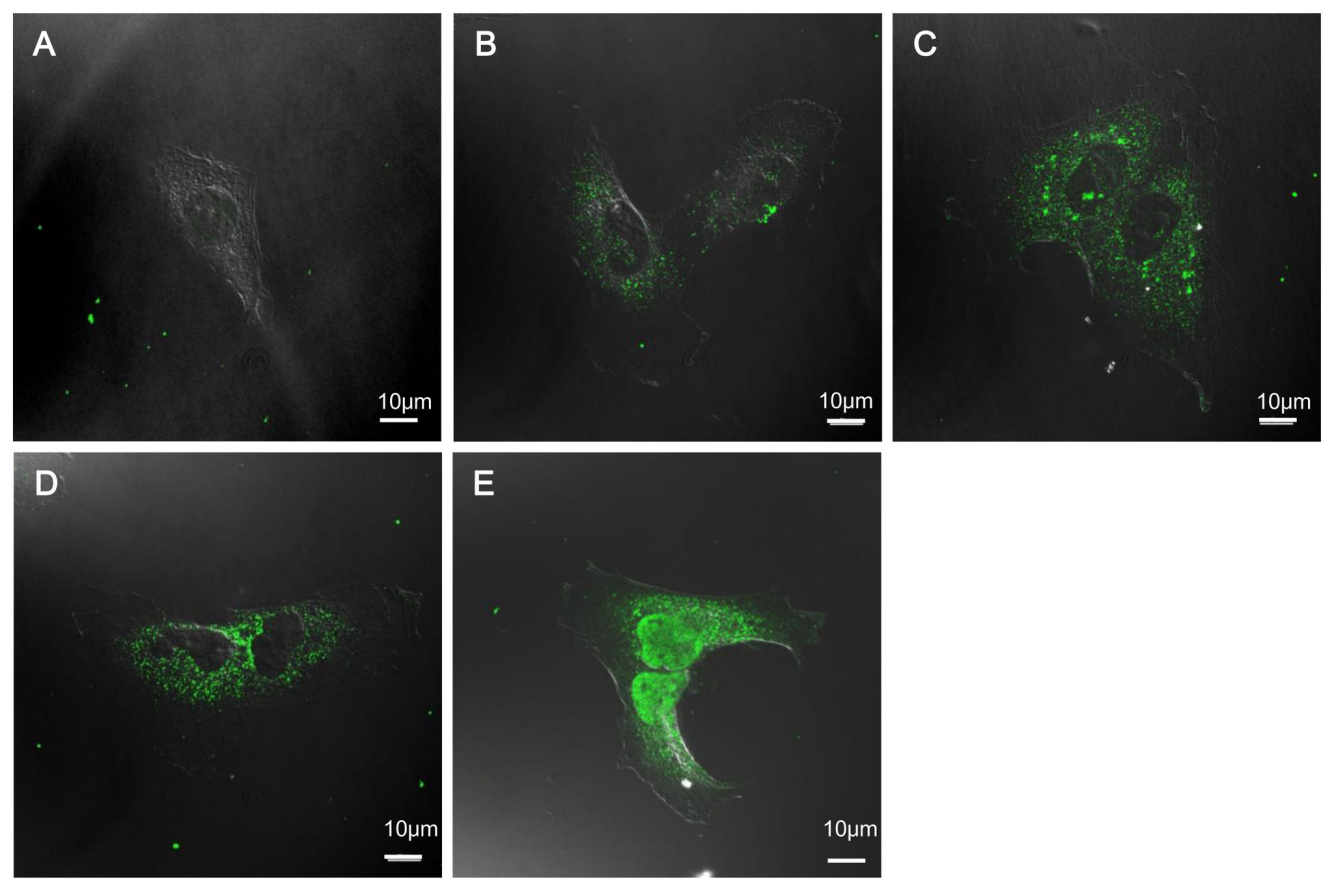
Cellular uptake of amelogenin. Time course confocal images of rM180 amelogenin internalization. After incubation at 4°C for 1 h, SaOS-2 cells were incubated with rM180 (30µg/mL) at 37°C for 0, 2, 5, 10, or 15 min, respectively (**A**-**E**). For fluorescence microscopy, the cells were stained with an anti-amelogenin antibody followed by an Alexa 488 secondary antibody (green): gray is the transmission image. Cells were visualized under the Nikon A1 fluorescence microscope using 63×/1.49 NA oil objectives. Images were obtained with the NIS-Elements AR 3.0 software, and the imaging parameters were kept constant whenever the intensity of fluorescence was to be compared. All confocal images are representatives of experiments conducted in triplicates. Scale bars: 10 µm.

To demonstrate the cellular uptake of amelogenin, rM180 (30 µg/mL) was added exogenously to SaOS-2 osteoblastic cells. Immunofluorescence cell imaging captured by confocal microscopy indicated that rM180 attached to the plasma membrane and internalized in the cells. To further validate the internalization process, SaOS-2 cells were treated for 30 min with rM180. Figure 2 shows the time course of rM180 uptake in SaOS-2 cells. rM180 was rapidly endocytosed in vesicles and localized around the perinuclear region within 15 min. No signal was observed in cells where the primary antibody was omitted.

### Identification of amelogenin-binding proteins in osteoblastic cells

Amelogenin proteins appeared to be internalized into the cells quickly, indicating that they are taken up by cells rather than binding to cell surface proteins such as cell surface receptors. As a first step to investigate the physiological role of amelogenin on osteogenesis, we tried to identify novel binding partners, which could be related to the hitherto unknown functions or mechanism of amelogenin in osteoblastic cells. Thus, we used a GST pull-down approach to capture endogenous binding proteins. GST-rM180 was incubated with protein extracts from SaOS-2 osteoblastic cells. SaOS-2 cells were separated into soluble (cytosolic) and membrane- associated (membrane) fractions to characterize the localization of amelogenin binding proteins. GST alone was used as negative control to subtract non-specific binding proteins. Cellular proteins interacting with GST-rM180 were then resolved by 2D-PAGE and identified by MALDI-TOF MS analysis.

Protein spots were selected for MS analysis by using the following criteria: (i) the proteins were reproducibly present in 5 independent experiments of Coomassie brilliant blue-stained gels of GST-rM180 + cell lysate compared with GST-rM180 alone and GST + cell lysate control gels and (ii) protein spots migrating at the same position in 2D gels of GST-rM180 + cell lysate as in 2D gels of pull-down with GST-rM180 only and GST control were subtracted from the list of protein spots and not considered for further identification.

**Figure 3:**
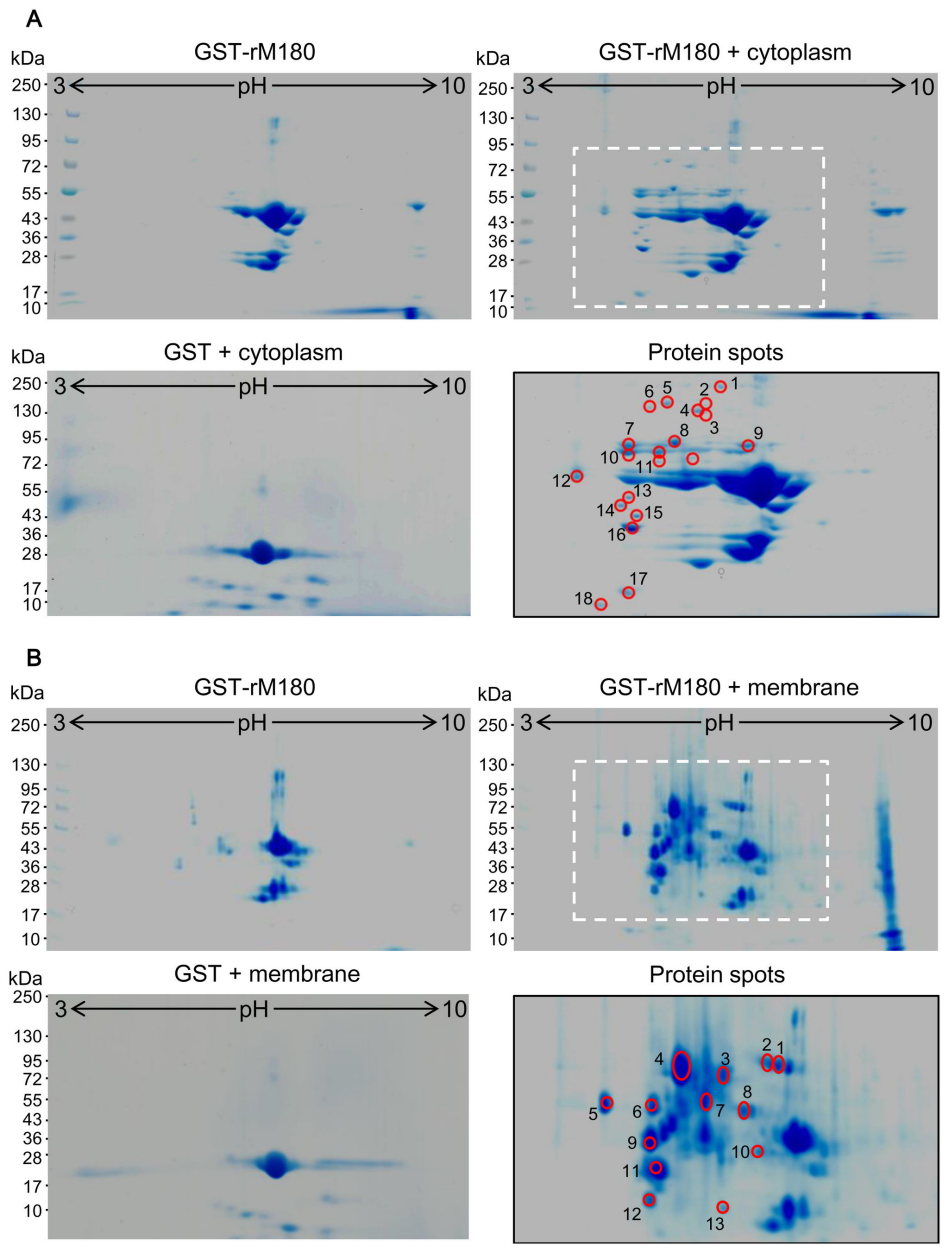
Proteomic analysis of amelogenin-interacting proteins in osteoblastic cells. Purified GST-rM180 immobilized on glutathione-Sepharose 4B beads was incubated with no extract (GST-rM180), fractionated soluble protein extract (GST-rM180 + cytoplasm) (**A**) or membrane-associated protein extract (GST-rM180 + membrane) (**B**) prepared from SaOS-2 cells. GST control gels for the both extracts ware also shown to exclude the possibility to non-specific bindings (GST + cytoplasm, GST + membrane). To minimize binding of nonspecific proteins, the cell extracts were pre-cleaned with glutathione beads. The proteins bound to affinity matrices were eluted and separated by isoelectric focusing and SDS-PAGE was performed on a 7.5–15% gradient gel. A typical two-dimensional gel is illustrated. The pH gradient of the separation in the first dimension is shown on the top of the gels, and the molecular weight markers are shown in kDa on the left of the gels. Proteins were visualized with Coomassie brilliant blue staining, excised, trypsinized, and analyzed by matrix-assisted laser desorption/ionization time-of-flight mass spectrometry (MALDI-TOF MS) analysis as described in Table **2**, 3. Magnified views of indicated areas were shown to demonstrate the analyzed spots of amelogenin-interacting proteins (Protein spots).

Figure 3 illustrates a typical gel for each condition. Importantly, no additional significant binding was detected when GST protein was incubated with cellular proteins. The GST-rM180 protein is represented as major spot at 50 kDa. There are several specific protein spots of smaller molecular weight, which likely represent breakdown products of GST-rM180. The list of identified proteins is shown in Table 2. A number of chaperone molecules such as heat shock proteins (HSPs) and glucose-regulated protein (Grp78/Bip), cytoskeletal proteins (actin, vimentin, tubulin) and actin-binding proteins (gelsolin, tropomyosin), a proton pump protein (ATPase), and sialic acid-binding Ig-like lectins (e.g., Siglec-10) were identified.

**Table 2:** Identification of amelogenin-interacting proteins in the soluble protein fraction of SaOS-2 cells.

**Spot protein identified^a^**		**Accession number^b^**	**Protein parameters (Theo.)^c^**		**Mascot score^d^**
			**Mo.wt**	**pI**	
			**(kDa)**		
1	gelsolin isoform b	gi|38044288	80.88	5.58	64
2	MTHSP75	gi|292059	74.02	5.97	75
3	No significant hits				
4	heat shock cognate 71 kDa protein isoform 1	gi|5729877	71.08	5.37	149
5	Grp78/BiP precursor	gi|386758	72.16	5.07	327
6	molecular chaperone DnaK (*Escherichia coli*)				
7	tubulin alpha-1B chain	gi|34740335	50.80	4.94	214
8	vimentin	gi|340219	53.74	5.03	100
9	tubulin alpha-1B chain	gi|34740335	50.80	4.94	207
10	beta-tubulin	gi|338695	50.24	4.75	132
11	tubulin, beta 2C, isoform CRA_b	gi|338695	49.25	4.88	165
12	actin, cytoplasmic 1	gi|4501885	42.05	5.29	172
13	tropomyosin alpha-4 chain isoform 2	gi|4507651	42.05	5.29	172
14	outer membrane protein F [*Escherichia coli* M605]				
15	No significant hits				
16	No significant hits				
17	myosin regulatory light chain 12A	gi|5453740	19.84	4.67	179
18	sialic acid binding Ig-like lectin 10	gi|119592421	47.74	9.41	54
19	ATPase beta	gi|226371	50.23	4.86	138
20	No significant hits				

a The numbers correspond to those illustrated in Figure **2A**.

b NCBI accession, accession number from the NCBI database of matched proteins

c Theo. Mr (kDa)/pI, the theoretical molecular mass and isoelectric point based on the amino acid sequence of the identified protein

d Mascot score, score obtained from Mascot for each match proteins

Next, we investigated the proteins in the membrane fraction to further identify the molecules involved in cellular uptake of amelogenin. To do so, a GST pull-down assay was also performed using membrane-associated protein extracts. Representative 2D images and proteins characterized by MALDI-TOF MS/MS analyses are shown in Figure 3B and Table 3. Several membrane-associated proteins were identified as amelogenin-binding partners. These include (i) endoplasmic reticulum (ER) protein (Grp78/Bip, calreticulin), (ii) mitochondrial membrane protein (prohibitin), and (iii) nuclear proteins: nucleolar protein, i.e., (nucleophosmin), heterogeneous nuclear ribonucleoprotein (hnRNP A2/B1). Interestingly, we identified the Grp78/Bip protein in both cytosolic and membrane-enriched fractions.

**Table 3:** Identification of amelogenin-interacting proteins in the membrane fraction of SaOS-2 cells.

**Spot protein identified^a^**		**Accession number^b^**	**Protein parameters (Theo.)^c^**		**Mascot score^d^**
			**Mo.wt**	**pI**	
			**(kDa)**		
1	caldesmon	gi|179830	62.72	6.18	102
2	unnamed protein product	gi|189054178	66.15	7.62	131
3	MTHSP75	gi|292059	74.02	5.97	86
4	Grp78/BiP	gi|6470150	71.00	5.23	187
5	calreticulin	gi|119604736	48.10	4.30	90
6	protein disulfide-isomerase precursor	gi|20070125	57.48	4.76	127
7	60 kDa heat shock protein, mitochondrial	gi|31542947	61.19	5.70	93
8	Chain A, TapasinERP57 HETERODIMER	gi|220702506	54.54	5.61	160
9	unnamed protein product	gi|16552261	47.52	5.01	136
10	Chia A, Crystal Structure Of Human Grp78/Bip	gi|320089786	42.28	6.00	103
11	nucleophosmin isoform 2	gi|40353734	29.62	4.47	121
12	oligopeptide/dipeptide ABC transporter, ATPase subunit	gi|344339629	29.62	4.47	104
13	prohibitin	gi|46360168	29.86	5.57	237

a The numbers correspond to those illustrated in Figure **2B**.

b NCBI accession, accession number from the NCBI database of matched proteins

c Theo. Mr (kDa)/pI, the theoretical molecular mass and isoelectric point based on the amino acid sequence of the identified protein

d Mascot score, score obtained from Mascot for each match proteins

### Validation of the interaction between amelogenin and Grp78/Bip

**Figure 4:**
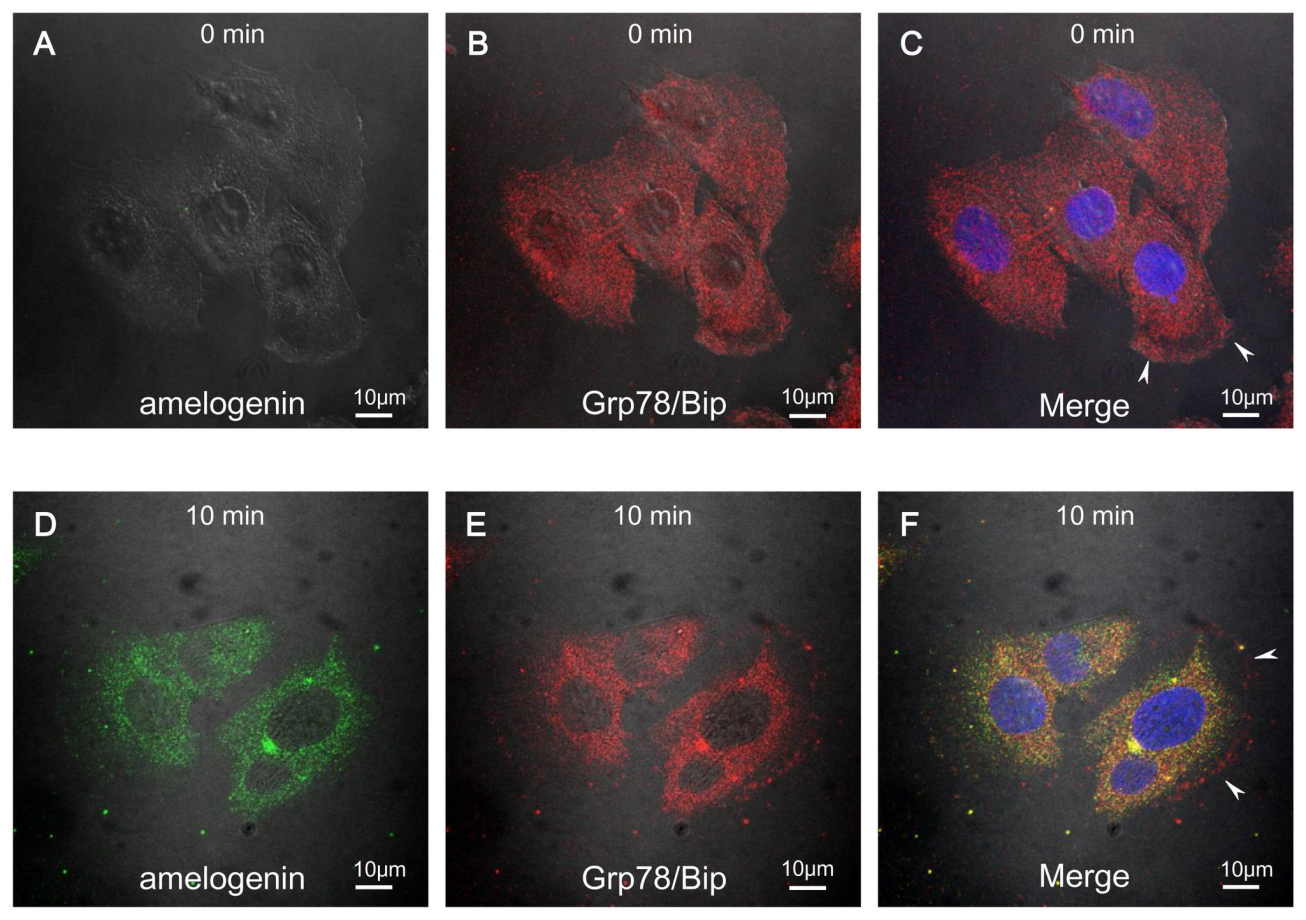
Grp78/Bip mediates cellular uptake of amelogenin. Co-localization of rM180 amelogenin and Grp78/Bip. After incubation at 4°C for 1 h, SaOS-2 cells were incubated with rM180 (30µg/mL) at 37°C for 10 min. For fluorescence microscopy, the cells were stained with amelogenin antibody (A and D; green) and Grp78/Bip antibody (B and E; red): gray is the transmission image. Nuclei were stained with Hoechst dye (blue). The co-localizatoion was illustrated in a merged image (**F**; yellow). Note that white arrowheads point to membranous localization of Grp78/Bip. Cells were visualized under the Nikon A1 fluorescence microscope using 63×/1.49 NA oil objectives. Images were obtained with the NIS-Elements AR 3.0 software, and the imaging parameters were kept constant whenever the intensity of fluorescence was to be compared. All confocal images are representatives of experiments conducted in triplicates. Scale bars: 10 µm.

To confirm the specificity of the interaction of amelogenin with Grp78/Bip, we first examined the co-localization of amelogenin and Grp78/Bip within the same cellular compartment of intact cells (Figure 4). We performed the current immunofluorescence co-localization studies in SaOS-2 cells. The localization of endogenous Grp78/Bip in the presence or absence of exogenously added rM180 was visualized under confocal laser scanning microscope. In the absence of rM180, Grp78/Bip was localized mainly in the cytoplasm (Figure 4C), as indicated by the previous observations. After the addition of rM180, majority of Grp/78 was concentrated in the perinuclear region, along with some in the plasma membrane rafts (Figure 4F). Consistent perinuclear localization of the endocytosed rM180 that co-localized with Grp78/Bip was observed. The significant co-localization of rM180 and Grp78/Bip suggested the *in vivo* association in SaOS-2 osteoblastic cells.

**Figure 5:**
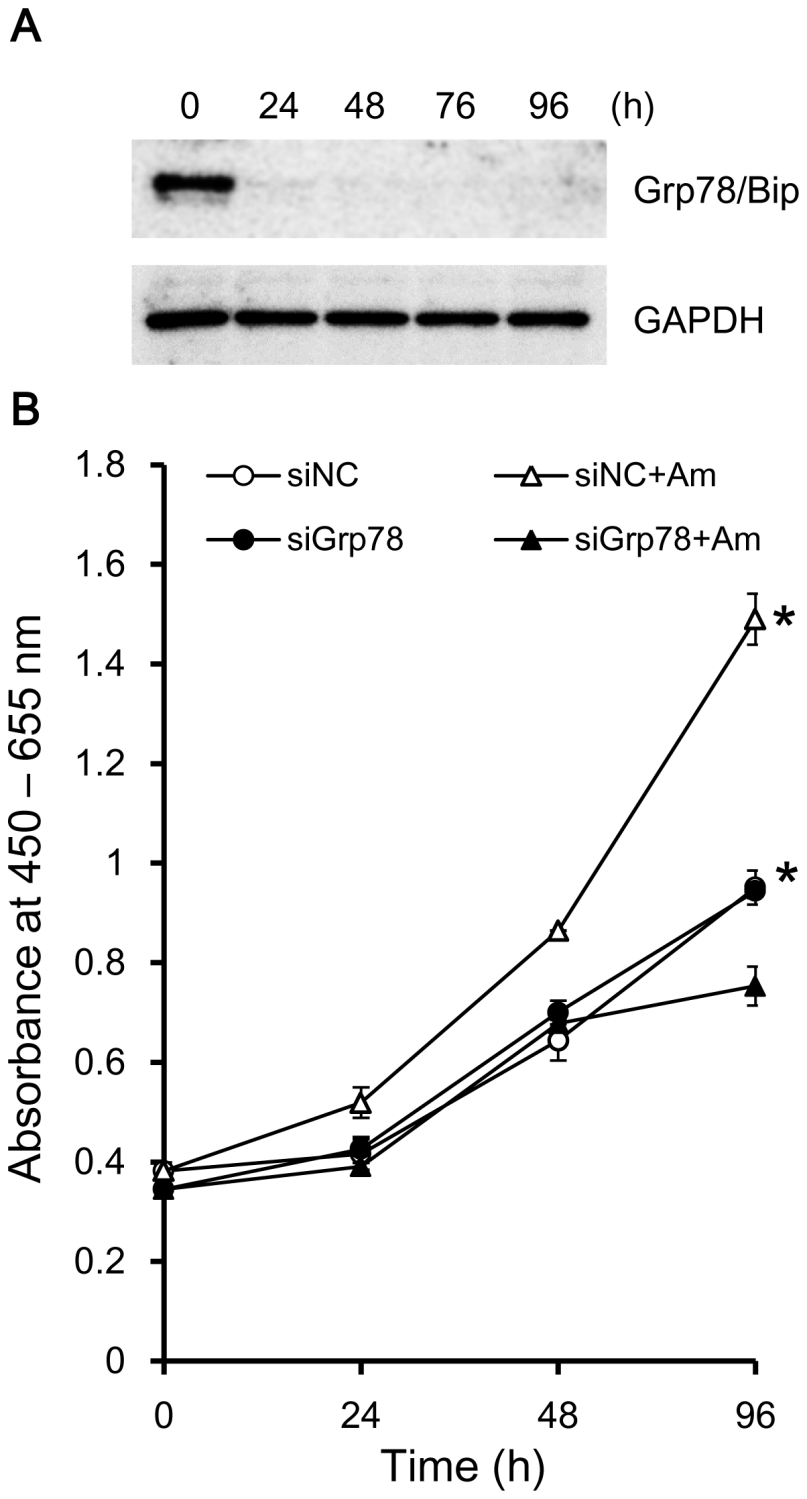
Knockdown of Grp78/Bip abrogated amelogenin-induced cell proliferation. **A**: Representative Western Bot analysis of Grp78/Bip siRNA-transfected SaOS-2 cells. SaOS-2 cells were transfected with Grp78/Bip siRNA, and total cell lysate was harvested at various time points following transfection and analyzed for indicated protein. GAPDH was used as a loading control. **B**: Proliferation rate of siRNA-mediated SaOS-2 cells. SaOS-2 cells were transfected with Negative Control (NC) or Grp78/Bip (siGrp78) siRNA incubated in growth medium (GM) with and without the addition of 30µg/mL rM180 (Am). WST-8 assay was performed for the indicated times. Proliferation is expressed as the absorbance at 450 nm minus the absorbance at 655 nm. Values are means ± S.D., *n* = 3. Statistical analyses were performed using a two-tailed unpaired Student's test, *, *p* < 0.01.

Next, we evaluated the potential biological effect induced by the interaction of amelogenin with Grp78/Bip. We assumed that Grp78/Bip is involved in amelogenin-induced proliferative regulation of osteoblasts. To determine if the association has a role in cell proliferation, we examined the effect of Grp78/Bip knockdown with and without the addition of rM180 in SaOS-2 cells (Figure 5). SaOS-2 transfected with nonspecific siRNA were used as control. The efficiency of the siRNA-mediated knockdown was confirmed by Western blot analysis (Figure 5A). Transfection with Grp78/Bip siRNA greatly reduced expression of Grp78/Bip protein. Addition of rM180 significantly increased the cell proliferation from 48 h through 96 h, compared with control cultures (Figure 5B). While the knockdown of Grp78/Bip had no effect on cell proliferation even with rM180, we observed that Grp78/Bip siRNA abrogated amelogenin-induced cell proliferation.

**Figure 6:**
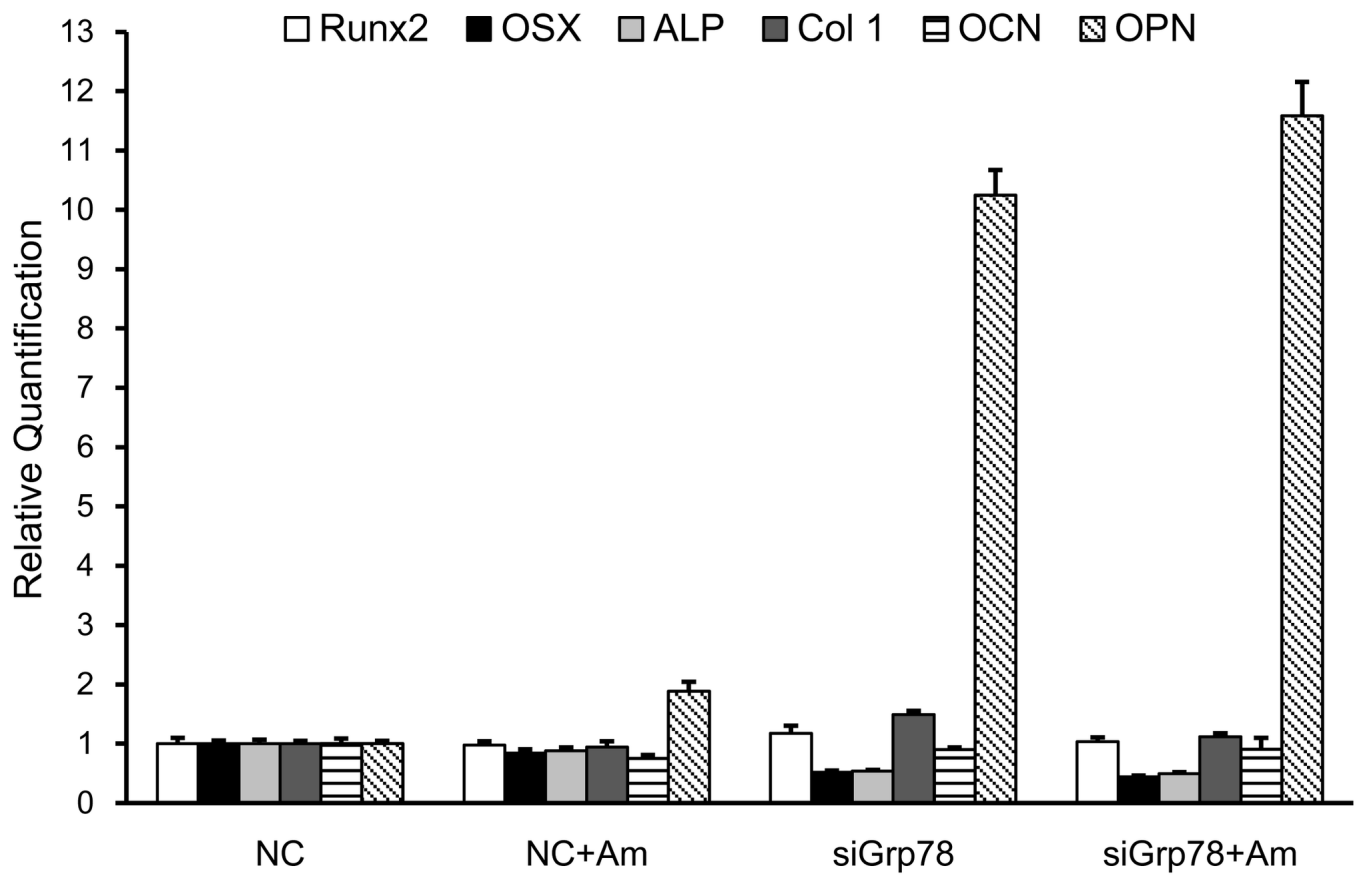
Effect of Grp78/Bip knockdown on osteoblastic marker expression during amelogenin stimulation. Negative Control (NC) or Grp78/Bip (siGrp78) siRNA transfected SaOS-2 cells were seeded at a density of 3 × 10^5^ cells / 24-well plate and incubated in growth medium (GM) for 24 h. After confirming the confluence of each wells, the cells were continuously incubated in osteogenic medium (OM) with and without the addition of 30µg/mL rM180 (Am) for 48 h. Total RNA was isolated and subjected to qRT-PCR analysis for the expressions of Runx2, Osterix (Osx), ALP, Type I collagen (Col 1), Osteocalcin (OCN), and Osteopontin (OPN). Expression was normalized to both house keeping genes β-actin and GAPDH and depicted as mRNA concentration relative to Negative Control (NC) siRNA-transfected cells. Values are means ± S.D., *n* = 3.

We further examined the downstream effect of Grp78/Bip knockdown in SaOS-2 cell on the expression of osteoblastic marker genes such as Runx2, Osterix (Osx), ALP, Type I collagen (Col 1) (early differentiation markers), Osteocalcin (OCN), and Osteopontin (OPN) (late differentiation markers) (Figure 6). rM180-induced expression of these marker genes was detected in SaOS-2 cells transfected with the control siRNA after 48 h. Quantitative real time RT-PCR on RNA samples showed that rM180 stimulation caused 2-fold increase in OPN mRNA expression at 48 h, but had little or no effect on other gene expression. Interestingly, the Grp78/Bip knockdown induced a significant 10-fold increase in OPN expression, although the expression of Osx and ALP was clearly suppressed, as expected from previous studies [26,27]. The knockdown did not suppress rM180-induced expression of OPN. Stimulation of rM180 on the Grp78/Bip knockdown had no effect on other genes. These results indicate that Grp78/Bip is necessary for amelogenin-induced cell proliferation, but not for osteogenic induction during the early differentiation period of SaOS-2 cells.

## Discussion

In this study, we identified new amelogenin-binding proteins in osteoblasts by combining affinity chromatography and proteomic analysis. As a precondition for the physiological interaction between amelogenin and cellular proteins, we clearly observed the internalization of amelogenin in osteoblasts. Other researchers reported that exogenously added amelogenin traffics to the perinucleus of the cells [28]. However, in our study, the nuclear accumulation of amelogenin was also observed via the perinuclear region. This may be because of different experimental conditions, the differences in the cell types used, or the fact that the cell density can affect the rate of endocytosis [29].

Previous research has shown that recombinant amelogenin can promote osteoblast differentiation [30]. In SaOS-2 osteoblastic cells, 16 proteins were identified as amelogenin-interacting proteins in the cytoplasm (Table 2). Among them were mainly cytoskeletal proteins, but also several chaperone molecules of HSP70 family proteins. Our findings that 9 of them are components of the cytoskeleton (actin, vimentin, tubulin), actin-binding proteins (gelsolin, tropomyosin), and a v-ATPase supports the notion that amelogenin may be bound to the cytoskeleton prior to activation. The significance of cytoskeletal organization for osteogenic differentiation and induction of mineralization has been reported [31,32]. The binding and modulation of v-ATPase to the actin cytoskeleton has previously been indicated [33], and the remodeling of the actin cytoskeleton is also controlled by an actin-binding protein, gelsolin [33]. Thus, the interaction with these cytoskeletal proteins suggests that amelogenin is involved in the differentiation process, possibly by regulating actin filament organization and dynamics.

HSP70 family proteins have been well characterized as molecular chaperons [34]. The association of these proteins suggests the assembly of a protein complex containing amelogenin. Because HSP70 family proteins have also been implicated in the regulation of signal transduction and apoptosis [35], it is also possible that amelogenin transmits certain intracellular signals , e.g., anti-apoptotic signals, through these molecules. Similarly, it was recently found that Siglec-10 selectively inhibits the immune response of damaged cells [36]. These findings supports the notion that amelogenin might regulate apoptosis-related signaling.

Next, we investigated the molecules involved in endocytosis pathways of amelogenin. The proteomic profiles of amelogenin-binding proteins in the membrane fraction were quite different from those of the cytosolic fraction. Many of them were ER-associated proteins (Grp78/Bip, protein-disulfide isomerase, calreticulin, and Tapasin-ERP57), whereas others were mitochondrial proteins (prohibitin, MTHSP75) and a nucleoprotein (nucleophosmin /B23). These ER-resident proteins contain at their carboxyl terminus the KDEL sequence, known to operate as signal for retention in the ER. However, these results are not surprising, because it has been reported that some ER-resident proteins, includingGrp78/Bip, calreticulin, and protein-disulfide isomerase, are not exclusively restricted to the lumen of the ER, but also expressed on the cell surface [37]. Furthermore, Xu et al. reported the co-localization of exogenous amelogenin and the ER [38], which report supports our current data. The ER plays a crucial role in protein biogenesis, signal transduction, and calcium homeostasis by being an intracellular calcium store [39]. As bone mineralization involves deposition of nascent calcium in form of hydroxyapatite crystals, intracellular calcium homeostasis is expected to play a vital role in the process of osteoblasts. Moreover, ER chaperones are widely known as a marker for ER stress, and recent studies have revealed that ER stress induced signaling promotes bone formation [26,27]. A likely model is that amelogenin by interacting with these proteins might block the KDEL sequence, thereby allowing them to leave the ER, which could cause ER stress. Therefore, binding with these ER-resident proteins could result in nascent polypeptide modification of proteins that may be important for amelogenin induced osteoblast mineralization [19], where maturation of matrix proteins is required.

It should be noted that Grp78/Bip was identified in both soluble and membrane protein fractions. Grp78/Bip has a trans-membrane domain that spans the ER and can therefore be seen in both membrane and lumenal cellular fractions [40]. Consequently, Grp78/Bip can bind to amelogenin in the membrane, cytosol, and lumen of the ER. The co-localization of amelogenin and Grp78/Bip around the perinuclear region (Figure 4) supports this notion. Interestingly, Grp78/Bip is reported to act as a cell surface receptor for DMP1 and translocated to the nucleus, resulting in osteoblast differentiation [41,42]. Furthermore, the expression level of Grp78/Bip was appeared to be involved in the development of mineralizing tissues including bone and teeth [43]. Grp78/Bip has also been identified to be cell surface signaling receptor for α2-macroglobulin in macrophages [44]. Thus, our findings strongly suggest that Grp78/Bip can be a candidate for the receptor of amelogenin. Previous studies have reported that while amelogenin is endocytosed via lysosome-associated membrane protein 1- and lysosome-associated membrane protein 3 (CD63)-positive vesicles, a specific receptor for amelogenin has not been identified [28,45]. Considering that lysosome-associated membrane protein 1 and CD63 are lysosome-associated proteins, the putative machinery for the endocytic trafficking of amelogenin could be explained with recent findings that suggest a direct involvement of the ER in the establishment of early lysosomal structures [46]. 

Interestingly, we observed that the interaction of amelogenin with Grp78/Bip contributed to cell proliferation, rather than the correlation with osteogenesis in SaOS-2 cells (Figures 5, 6). Grp78/Bip is a stress-inducible protein that is ubiquitously expressed in eukaryotic cells. Grp78/Bip knockout mice exhibit embryonic lethality, indicating that Grp78/Bip is essential for cell growth [47]. The expression of Grp78/Bip is strongly up-regulated in many cancer cells, while it is maintained at a low basal level in normal cells [48]. In our confocal microscopy-based co-localization analysis, we observed that a subpopulation of Grp78/Bip was localized on the plasma membrane of SaOS-2 cells. Recent studies have demonstrated that the preferential expression of Grp78/Bip on cancer cell surface plays critical roles in cell signaling, proliferation, and survival [49]. Considering that SaOS-2 cells are osteosarcoma cell lines as well as osteoblastic cells, it is appropriate that the interaction of Grp78/Bip with amelogenin was associated with amelogenin-induced cell proliferation [13]. Earlier studies support the fact that EMD affects the early states of osteoblastic maturation by stimulating proliferation, but as cells mature, the differentiation is enhanced by EMD [50]. Similarly, human recombinant basic fibroblast growth factor (FGF-2), which is under multi-center clinical trials in Japan for periodontal tissue regeneration, is considered to promote the proliferation of osteoblasts rather than to induce differentiation [51]. In support of the notion that SaOS-2 cells exhibit early-stage features of osteoblastic cells, osteogenic gene expression analysis showed that SaOS-2 cells closely resemble primary osteoblasts [52].

Another interesting finding of this study was that Grp78/Bip knockdown induced significantly high OPN expression regardless of the presence or absence of rM180.　 Contrary to the previous studies that ER stress elevates Grp78/Bip expression and thereby induces osteoblast differentiation, the suppression of Osx and ALP was predictable from this study [26]. OPN had been originally identified as a transformation-associated protein [53], and contributes to tumorigenesis and metastasis [54]. Recent proteomic approach suggests OPN as a biomarker of hepatocellular carcinoma (HCC) [55], and Grp78/Bip knockdown enhanced cell migration without contributing to cell proliferation in HCC [56]. Our observations in SaOS-2 cells are supported by these previous reports. Further studies are needed to validate the osteogenic effects induced by amelogenin-Grp78/Bip interaction in different stage of osteoblast formation. To the best of our knowledge, this is the first study showing evidence of biological interactions between amelogenin and Grp78/Bip.

In conclusion, our proteomic approach provided significant understanding of amelogenin interaction networks. Amelogenin interactions between individual proteins have been identified in both soluble and membrane fractions of SaOS-2 osteoblastic cells. Although the biological significance of most of these interactions remain to be explored, our work represents a key step toward understanding the biological components and their roles involved in the signal transduction underlying osteoblast differentiation and their function in bone regeneration. Finally, we determined the interaction of amelogenin with Grp78/Bip. Our findings are of considerable therapeutic significance and help improve our understanding of the cellular and molecular bases of amelogenin-induced periodontal tissue regeneration.
